# Lost and Found: A Case Report of the Journey of Two Teeth Into the Bronchus After a Road Tragedy

**DOI:** 10.7759/cureus.64622

**Published:** 2024-07-15

**Authors:** Sakshi S Dudhe, Gaurav V Mishra, Pratapsingh Parihar, Devyansh Nimodia, Saurav S Dudhe, Anjali Kumari, Dhanajay Shinde

**Affiliations:** 1 Radiodiagnosis, Datta Meghe Institute of Higher Education and Research, Wardha, IND; 2 Medicine, All India Institute of Medical Sciences, Nagpur, IND

**Keywords:** foreign body airway obstruction, trauma, tooth, teeth, bronchus, foreign body

## Abstract

Traumatic foreign body aspiration (FBA) in adults is a rare yet potentially life-threatening event that requires prompt recognition and management to prevent adverse outcomes. While less common in adults compared to paediatric populations, traumatic FBA incidents often occur in occupational settings, post-trauma, or during high-risk activities, presenting with acute respiratory symptoms and varying degrees of airway obstruction. Diagnosis can be challenging due to the lack of classic symptoms and the potential for delayed presentation, necessitating a thorough history, a focused physical examination, and appropriate imaging techniques such as computed tomography (CT) and bronchoscopy. Prompt intervention is crucial to mitigating complications such as airway obstruction, pneumothorax, and respiratory compromise. Here, we describe an interesting case of a patient with a road traffic accident who aspirated two teeth and thought he lost them in this process. Surprisingly, two lost teeth were found after undergoing diagnostic procedures for mild shortness of breath, further causing aspiration pneumonitis.

## Introduction

Teeth are an infrequent and rare object among the wide range of objects that can become trapped in the airways. Tooth aspiration into the bronchus usually results from traumatic events, including falls or accidents, where respiratory compromise and tooth damage happen simultaneously. Aspirating a tooth into the bronchus increases the risk of tissue damage, infection, and airway obstruction. Timely identification and management are essential to reducing these hazards and guaranteeing positive patient experiences. Although incidences of foreign body aspiration (FBA) are more frequently linked to paediatric populations (80% of cases), cases affecting adults emphasize the importance of being vigilant in all age groups. Clinical manifestations of FBA are discussed in detail, including common symptoms such as coughing, choking, dyspnoea, and wheezing. Diagnosing FBA in adults can present a challenge due to the potential lack of specificity in symptoms and the possibility that imaging studies may not consistently provide conclusive evidence of aspiration [1].

FBA, although rare, poses a potential threat to life and is responsible for a small percentage, ranging from 0.16% to 0.33%, of bronchoscopic procedures in adults. The manifestation of adult FBA commonly involves a choking episode followed by persistent coughing; however, it can sometimes resemble more persistent conditions such as chronic obstructive pulmonary disease (COPD), asthma, and obstructive pneumonia in cases where the initial incident is overlooked, particularly in elderly individuals with altered mental status [2]. Timely intervention plays a crucial role in attaining positive results and reducing the morbidity and mortality linked to tracheobronchial foreign bodies. Various complications, such as pneumonia and lung abscesses, have the potential to manifest over an extended period of several months [3].

Multidetector computed tomography (MDCT) likely enhanced the precision of diagnosis through the visualization of abnormalities in the airway and the identification of both the location and properties of the foreign object. This methodology probably resulted in a more focused and efficient approach to bronchoscopic procedures, thereby decreasing the occurrence of procedural complications and augmenting the outcomes for patients. MDCT plays a crucial role in the diagnostic process for suspected cases of FBA in patients, ultimately optimizing clinical treatment and enhancing the quality of patient care. The utilization of pre-bronchoscopy MDCT in cases involving suspected FBA is deemed to be highly advantageous. It is likely that they used MDCT to improve diagnostic precision by uncovering abnormalities in the airway and accurately identifying the location and features of the foreign object. This likely leads to more precise and effective bronchoscopic interventions, reducing procedural risks and improving patient outcomes. Flexible bronchoscopy is identified as the preferred method for both the diagnosis and removal of foreign bodies, offering direct visualization of the airway and the ability to retrieve the foreign object under direct vision [4]. Flexible bronchoscopy has been recognized as the optimal approach for both the diagnosis and retrieval of foreign bodies, providing a direct view of the airway and the capacity to recover the foreign entity while under direct observation [5]. Bronchoscopy plays a pivotal role as the preferred standard for both diagnosis and treatment in clinical practice.

## Case presentation

We present the case of a 51-year-old man, a driver by occupation, who met with a road traffic accident four months ago and sustained mild abrasions over the face. He could not seek any medical care for four months and subsequently presented with complaints of dry cough, mild shortness of breath, low-grade fever, and fatigue on and off for four months. His past history was a sign of hypertension. He had also been prescribed antibiotics for fever and cough.

**Figure 1:**
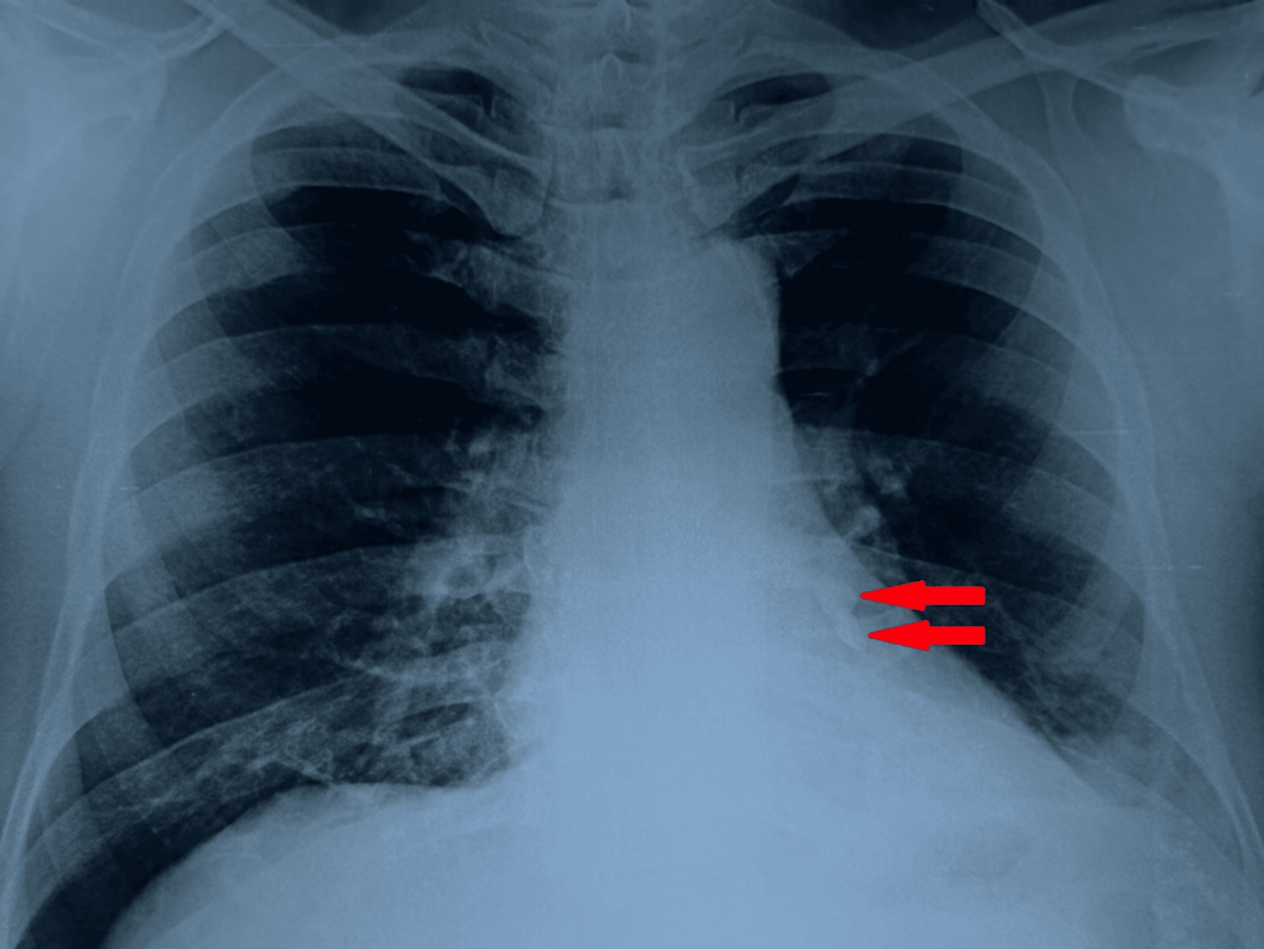
A chest radiograph showing two well-defined radiodense opacities in the left lower bronchus (red arrows).

Upon emergency department evaluation, the patient complained of mild left-sided chest pain, exacerbated by activity. Vital sign readings showed a heart rate of 110/min, a respiratory rate of 25/min, a blood pressure of 140/80 mmHg, an axillary temperature of 36.5 °C, and an SpO_2_ of 97%. Systemic respiratory system examination revealed decreased breath sounds over the left lung field with low-grade wheezing and stridor. The rest of the systemic examinations, such as the cardiovascular system, central nervous system, and gastrointestinal system, were within normal limits. The laboratory workup was unremarkable. A dental exam revealed two missing molars. A chest radiograph was advised, which showed two well-defined radiodense opacities noted in the left lower bronchus (Figure 1).

**Figure 2:**
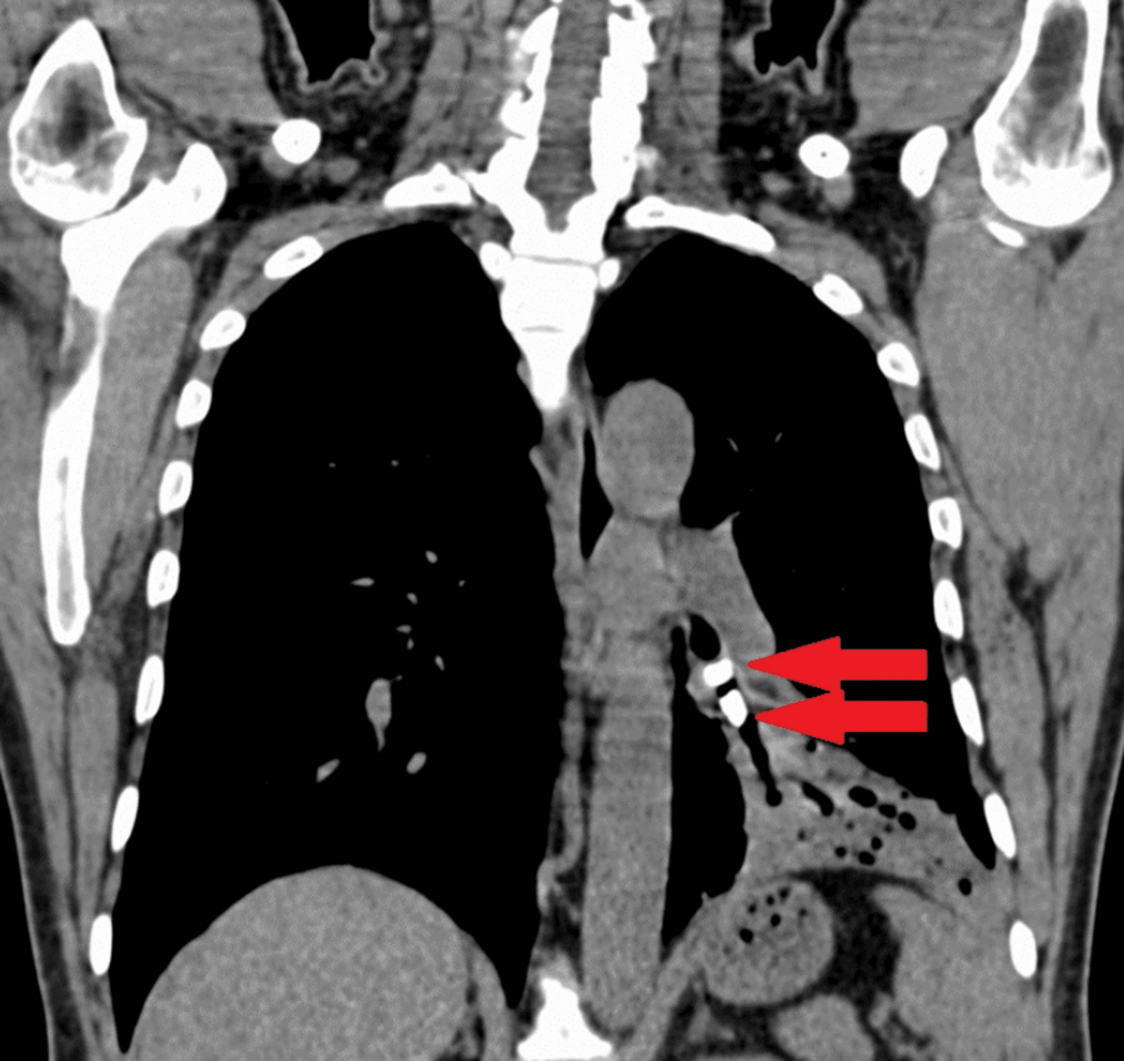
High-resolution computed tomography of the chest mediastinal window coronal section revealed two well-defined bone density areas (HU+1850 to +1980) seen within the left lower lobe bronchus, one measuring 11 x 8 mm and the other measuring 8 x 6 mm (red arrows).

**Figure 3:**
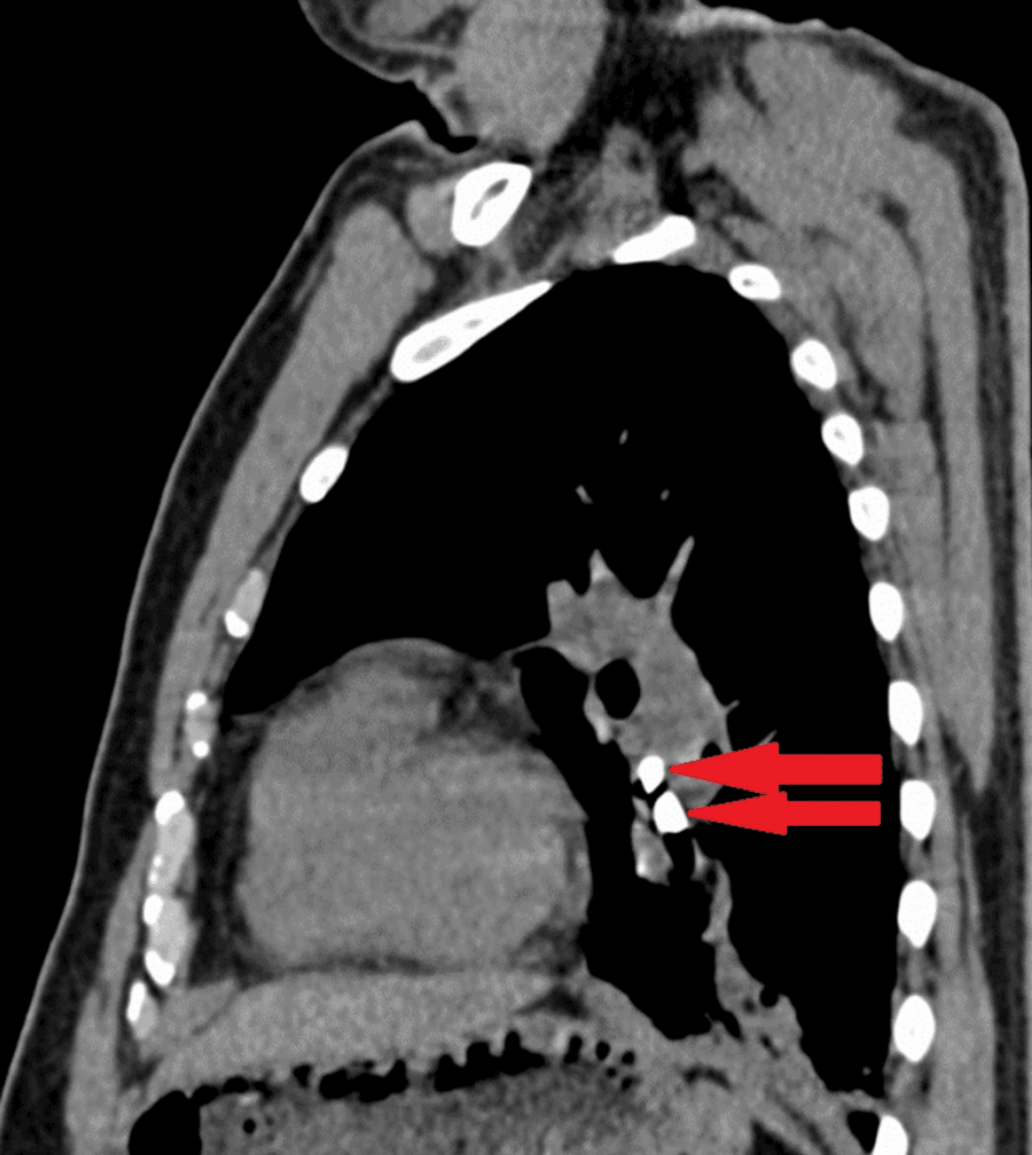
High-resolution computed tomography of the chest mediastinal window sagittal section revealed two well-defined bone density areas (HU+1850 to +1980) seen within the left lower lobe bronchus, one measuring 11 x 8 mm and the other measuring 8 x 6 mm (red arrows).

A high-resolution computed tomography (HRCT) of the chest revealed two well-defined bone density areas seen within the left lower lobe bronchus, one measuring 11 x 8 mm and the other measuring 8 x 6 mm (Figures 2, 3).

**Figure 4:**
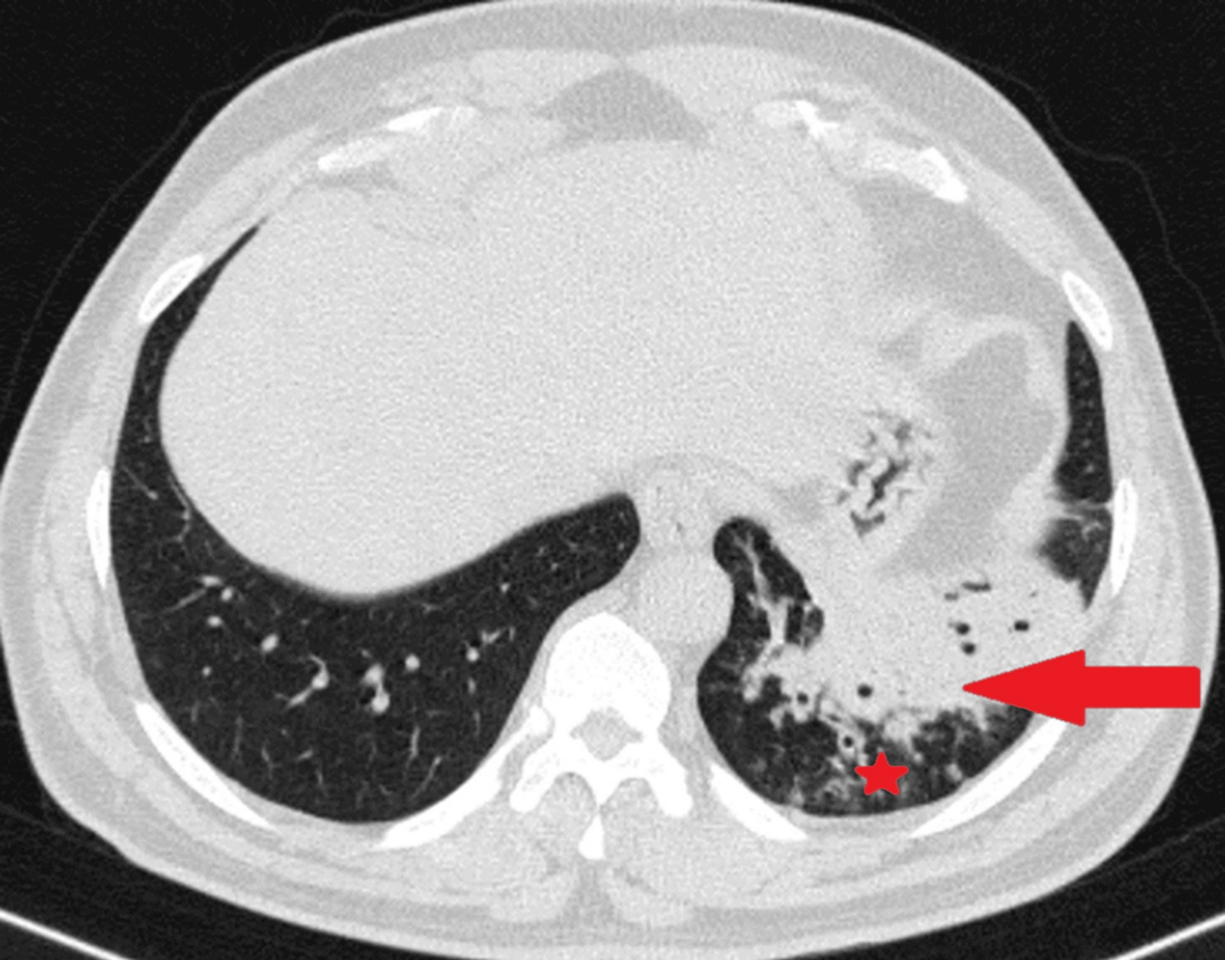
High-resolution computed tomography of the chest lung window axial section showed consolidation in the left lower lobe segments (red arrow) and a tree-in-bud appearance (star), representing aspiration pneumonitis.

Resultant consolidation has occurred in the antero-medial, postero-basal, and latero-basal segments of the left lower lobe, which is compatible with the two teeth that went missing. The presence of a tree-in-bud appearance and consolidation noted in the left lower lobe was indicative of aspiration pneumonitis (Figure 4).

Bronchoscopy revealed a partial endobronchial obstruction in the left lower lobe bronchus, caused by two teeth, with chronically inflamed surrounding mucosa. Bronchoalveolar lavage yielded negative bacterial and fungal cultures. Two days later, bronchoscopic retrieval of the two teeth was done. He was discharged on oral antibiotics. At follow-up, the patient reported that he had been symptom-free post-procedure.

## Discussion

A physical examination of a patient suspected of FBA might exhibit normal results or reveal nonspecific symptoms such as cough and hoarseness, particularly in cases involving small foreign bodies. Despite the spontaneous expulsion of the foreign body, these clinical manifestations may persist for several days. Symptoms exhibited by individuals with a foreign body lodged in the trachea or mainstem bronchi include stridor, chronic cough, significant dyspnoea, pronounced wheezing localized to the impacted side, or the absence of breath sounds on the affected side. Wheezing on the dependent side may be elicited by auscultation while the patient is in lateral decubitus positions. Due to the ball-wall effect, hyperinflation on the side where the foreign body is situated can lead to an enlarged appearance of the chest cage on that side. Conversely, total airway blockage caused by a foreign body leading to atelectasis may manifest as the absence of breath sounds on the impacted side.

Timely intervention in cases of FBA is essential to avert potentially severe medical consequences, such as the complete blockage of the respiratory passages [6]. FBA in adults is less prevalent than in children and may manifest with nonspecific symptoms such as cough, dyspnoea, or haemoptysis [7]. The diagnosis can be difficult in certain instances since the patient does not remember the aspiration incident. Irreversible lung damage and recurrent pneumonia, which might result from delays in diagnosing and treating these patients with traumatic injuries, have been reported.

Coughing, wheezing, or respiratory distress are helpful signs in addition to trauma-related ones. Trauma-related aspiration is a differential that should not be neglected when other traumatic changes, such as a rib fracture or pneumothorax, have been ruled out. Details about the time frame when the tooth was aspirated are crucial [8]. Irreversible lung damage and recurrent pneumonia might result from delays in diagnosing and treating these patients. A few cases of aspirated teeth in patients with traumatic injuries have been reported, and this case is one of them. A comprehensive evaluation of patients presenting with respiratory symptoms must be conducted if there is a mismatch between clinical observations and imaging to ensure that FBAs are not missed [9]. The size of the aspirated object is a single factor that can significantly impact a patient's clinical course in FBAs. Larger foreign bodies typically lead to wheezing, stridor, and coughing up immediately [10].

To confirm the diagnosis and locate the foreign body, a thorough history, physical examination, and suitable imaging techniques such as chest X-rays, computed tomography (CT) scans, or bronchoscopic evaluations are required. For the aspiration of foreign bodies to be properly managed, prompt intervention is necessary since consequences, such as pneumonia and lung abscesses, might take months to manifest [3]. In cases of unexplained pneumonia, particularly in atypical or rare presentations, FBA should be investigated as a differential diagnosis to enable quick diagnosis and action to enhance outcomes and prevent complications. Patients of all age groups can be affected by tooth aspiration, while it is more frequently linked to loose teeth, maxillofacial operations, or trauma. Aspirations of foreign bodies are significantly more common in those with neurological disorders [11]. The general population's awareness must be raised even more due to the substantial danger of morbidity and death from FBA [12].

CT imaging has provided valuable insights into the anatomy of the airways and the localization of foreign bodies, thus assisting in the planning of preoperative procedures. Moreover, bronchoscopy is often considered the preferred method for diagnosing FBA and enables direct visualization and extraction of the objects. The research suggests that the combination of CT imaging and bronchoscopy offers a comprehensive approach to diagnosing and managing FBA, ultimately enhancing patient outcomes and guiding clinical decision-making. Specifically, CT imaging offers essential preoperative information through detailed visualization of airway structures, aiding in the detection and precise localization of potential foreign bodies. Additionally, CT scans can reveal key indicators of FBA, such as hyperinflation, atelectasis, or focal air trapping, assisting in identifying affected lung segments and guiding the bronchoscopic procedure. Bronchoscopy is likely to confirm the presence of foreign bodies, evaluate their characteristics such as size, shape, and location, and facilitate their immediate retrieval, thereby reducing the risk of complications such as airway blockage or lung damage.

The importance of clinical suspicion and the predominant role of bronchoscopy as the preferred method for identifying and managing FBA are of great significance. Factors such as the type, size, and location of the foreign body, as well as the timeliness of intervention, play crucial roles in determining procedural success and patient outcomes. Additionally, post-bronchoscopy recovery is vital, encompassing symptom resolution, lung function improvement, and potential long-term consequences.

Various management techniques exist for treating tracheobronchial foreign bodies, ranging from bronchoscopic retrieval methods to surgical procedures and conservative approaches. Complications associated with these foreign bodies include airway blockages, pneumonia, and bronchial injuries. The advancement of standardized protocols, diagnostic technologies, and public education strategies are essential in addressing tracheobronchial foreign body cases. Collaboration among multidisciplinary teams and the implementation of standardized management protocols are believed to play key roles in reducing complications and optimizing patient outcomes.

## Conclusions

In conclusion, this case report highlights the importance of considering unusual etiologies when evaluating patients presenting with respiratory symptoms. The illustration in this particular case reveals how an aspirated tooth lodged in the bronchus can imitate respiratory ailments such as pneumonia or bronchitis, resulting in diagnostic complexities and potential treatment delays. Prompt recognition and intervention are crucial to prevent complications such as recurrent infections, abscess formation, or airway obstruction. Additionally, this case underscores the significance of thorough history-taking, physical examination, and utilization of imaging modalities for accurate diagnosis and timely treatment. Multidisciplinary collaboration between pulmonologists, otolaryngologists, and radiologists is essential for successful management and optimal patient outcomes. Early recognition of aspirated foreign bodies, including teeth, can lead to prompt retrieval and resolution of symptoms, thus minimizing morbidity and improving patient quality of life.

This case study accentuates the necessity of maintaining a vigilant attitude towards unusual origins of respiratory distress to ensure holistic patient management and positive clinical results. Post-traumatic manifestations such as cough, dyspnoea, wheezing, and occasionally haemoptysis raise suspicion of FBA and warrant regular monitoring and assessment for suspected aspiration. The potential for an extended asymptomatic progression or mild respiratory manifestations, such as cough and haemoptysis, as illustrated in our case, highlights the necessity for regular surveillance and assessment for aspiration. In instances where concerns arise regarding the potential displacement of foreign bodies into the airways, prompt imaging evaluation is essential. If detected, bronchoscopic retrieval should be pursued to prevent recurrent pneumonia and irreversible pulmonary injury.
